# Miniature Amphibious Robot Actuated by Rigid‐Flexible Hybrid Vibration Modules

**DOI:** 10.1002/advs.202203054

**Published:** 2022-08-18

**Authors:** Dehong Wang, Yingxiang Liu, Jie Deng, Shijing Zhang, Jing Li, Weiyi Wang, Junkao Liu, Weishan Chen, Qiquan Quan, Gangfeng Liu, Hui Xie, Jie Zhao

**Affiliations:** ^1^ State Key Laboratory of Robotics and System Harbin Institute of Technology Harbin 150001 China

**Keywords:** amphibious robot, friction locomotion, rigid‐flexible hybrid module, vibration‐induced flow

## Abstract

Amphibious robots can undertake various tasks in terrestrial and aquatic environments for their superior environmental compatibility. However, the existing amphibious robots usually utilize multi‐locomotion systems with transmission mechanisms, leading to complex and bulky structures. Here, a miniature amphibious robot based on vibration‐driven locomotion mechanism is developed. The robot has two unique rigid‐flexible hybrid modules (RFH‐modules), in which a soft foot and a flexible fin are arranged on a rigid leg to conduct vibrations from an eccentric motor to the environment. Then, it can run on ground with the soft foot adopting the friction locomotion mechanism and swim on water with the flexible fin utilizing the vibration‐induced flow mechanism. The robot is untethered with a compact size of 75 × 95 × 21 mm^3^ and a small weight of 35 g owing to no transmission mechanism or joints. It realizes the maximum speed of 815 mm s^–1^ on ground and 171 mm s^–1^ on water. The robot, actuated by the RFH‐modules based on vibration‐driven locomotion mechanism, exhibits the merits of miniature structure and fast movements, indicating its great potential for applications in narrow amphibious environments.

## Graphical abstract

A miniature amphibious robot actuated by dual rigid‐flexible hybrid modules is developed. It adopts the vibration‐driven mechanism for amphibious motion, with running motion by the friction locomotion mechanism and swimming motion by the vibration‐induced flow mechanism. The robot achieves a compact size with the integration of control system for no transmission mechanisms.

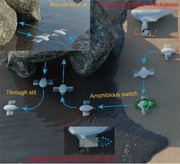

## 1 Introduction

Amphibious robots have unique advantages in the circumstances of environmental reconnaissance, information monitoring, resource exploration, and disaster relief^[^ ^1^, ^2^, ^3^ ^]^ due to their superior adaptability to complex terrestrial and aquatic environments. According to different locomotion mechanisms, the existing amphibious robots can be generally divided into legged amphibious robots,^[^ ^4^, ^5^, ^6^ ^]^ wheeled amphibious robots,^[^ ^7^, ^8^ ^]^ spherical amphibious robots,^[^ ^9^, ^10^ ^]^ tracked amphibious robots,^[^ ^11^, ^12^ ^]^ snake‐shaped amphibious robots^[^ ^13^, ^14^ ^]^ and so on.^[^ ^15^, ^16^ ^]^ Since the amphibious robots need to hold the ability to realize dual locomotion modes adapting to different environments, they are usually equipped with two types of locomotion systems or utilize complex transformable structures. Currently, the combination of different terrestrial and aquatic locomotion mechanisms provides a successful pattern to realize amphibious motion, but some disadvantages of each original mechanism are always inherited. Moreover, the associated transmission mechanism and switching structure between different locomotion systems are usually needed for the combination idea, leading to problems of complicated control scheme, complex structure, and difficulty in miniaturization.^[^ ^17^ ^]^ Most amphibious robots are over 200 mm in length by now, while some typical micro amphibious robots are achieved via special materials but the control system cannot be integrated.^[^ ^18^, ^19^, ^20^ ^]^ Amphibious robots of small sizes are necessary in some cases such as the exploration of narrow passages or caves in the swamp or coast environments. Thus, an integrated amphibious locomotion mechanism to simplify the structure and achieve the miniaturization is urgently required for the amphibious robots.

Vibration‐driven locomotion mechanism allows for flexible structural design and is convenient to realize a compact structure for the advantage of no transmission mechanism. The vibration‐based micro‐robots can be realized by piezoelectric actuators, dielectric elastomer actuators, or electromagnetic actuators. The first two actuators usually require complex excitation source to generate specific signal with high voltage or high frequency,^[^ ^21^, ^22^, ^23^, ^24^ ^]^ which makes it difficult to achieve power integration and restricts the miniaturization. The electromagnetic actuators can be directly powered by low‐voltage supply, thus it is convenient to be integrated and further realize a large motion range.^[^ ^25^, ^26^, ^27^, ^28^ ^]^ Some products have already been commercialized, such as HexBugs (https://www.hexbug.com/nano, Innovation First International, Greenville, Texas). The existing electromagnetic actuated robots mostly adopt inertial locomotion mechanism of eccentric motors, in which an inertial mass with periodic motion is used to achieve the locomotion for the asymmetric resistance with environment. However, there is usually a backward motion in one period, so that the efficiency is not satisfying, and the speed is not fast. Furthermore, the vibration‐driven mechanism is mainly used for terrestrial micro‐robots currently, whereas effective vibration‐based aquatic locomotion mechanisms are still rare. Some oscillation based aquatic robots on water surface^[^ ^29^, ^30^ ^]^ have been developed recently, but these locomotion mechanisms cannot combine with the existing terrestrial locomotion mechanism conveniently. According to the existing works, vibrations in fluid can induce directional movement of the fluid clusters, leading to the generation of flow.^[^ ^31^ ^]^ This vibration‐induced flow phenomenon is rarely used but has great potential for vibration‐based aquatic locomotion. It can be induced by the longitudinal vibrations such as the acoustic stream formed by the dissipation of sound wave,^[^ ^32^, ^33^, ^34^ ^]^ while it can also be induced by the transverse vibrations such as the flows appeared with the Faraday waves^[^ ^35^ ^]^ when fluid vibrates in vertical direction.

**Figure 1:**
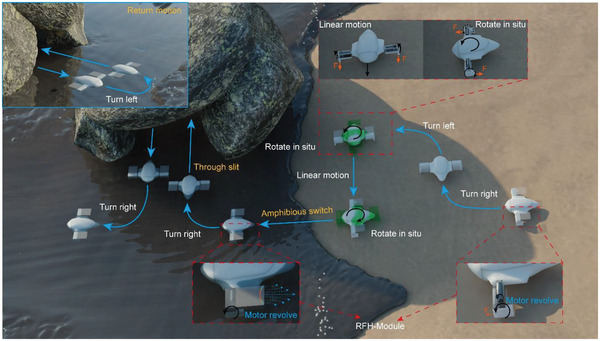
The motion scene diagram of the amphibious robot.

In this work, we design a unique rigid‐flexible hybrid module (RFH‐module) for achieving the amphibious motions by the vibration‐driven locomotion mechanism. The RFH‐module, adopting an eccentric motor as core unit, can excite the soft foot and flexible fin to realize dual locomotion modes of running and swimming, respectively. As for terrestrial motion, it adopts the friction locomotion mechanism, similar to our previous vibration‐driven crawling robot,^[^ ^26^ ^]^ the eccentric motor can generate elliptical vibration on the soft foot, then crawling motion on the ground is realized by frictional force. As for aquatic motion, we investigate the vibration‐induced flow mechanism of flexible plate on water surface and propose a vibration‐based aquatic locomotion method inspired by the directional flow of vibrating block and the movement of honeybees.^[^ ^31^, ^36^ ^]^ This RFH‐module offers significant advantages in achieving a miniaturized and hermetic design of amphibious robots, for the vibration‐driven principle does not require transmission mechanism or joints. The robot prototype achieves the integration of control system with size of 75 × 95 × 21 mm^3^ and weight of 35 g, and it can realize multiple forms of untethered motion in amphibious environments through the cooperation of two RFH‐modules. Thus, for the advantages of small size and flexible locomotion modes, it can serve for the exploration of small caves and slits, as shown in **Figure** **1**. In the following parts, the structure design, amphibious locomotion mechanisms, and motion characteristics of the robot will be described and discussed in detail.

## 2 Structure Design

**Figure 2:**
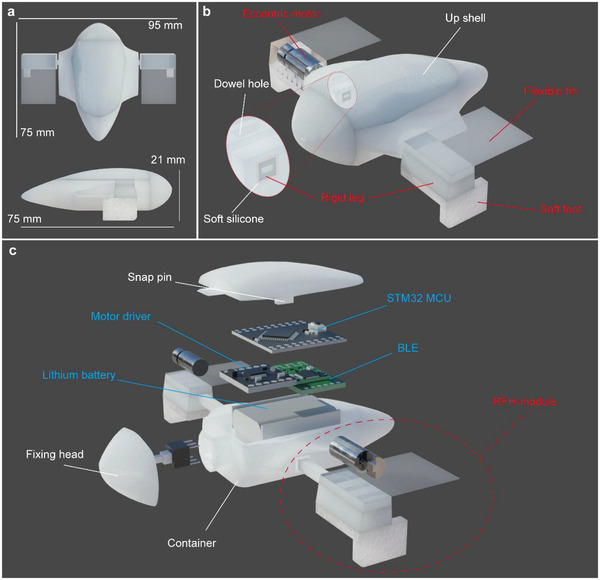
Configuration of the amphibious robot. a) The main sizes of the robot. b) The appearance and the vibration isolation structure at the connection between the RFH‐module and main body. c) The detail internal structure.

The appearance of the amphibious robot is designed in reference to the stingray, and the overall size is ≈75 mm × 95 mm × 21 mm, as shown in **Figure** **2**a. The robot has one main body and two locomotion modules, as shown with appearance view in Figure 2b and exploded view in Figure 2c. The main body is designed to be streamlined to reduce the resistance in water, and it consists of only three components (up shell, fixing head, and container) to facilitate the assembly process. It relies on two pairs of snap pins and dowel holes for positioning and uses the fixing head for fastening. All the outer gaps are filled with waterproof adhesive for sealing. The pair of RFH‐modules for amphibious locomotion is mounted on two sides of the robot, which are the core components to generate the driving forces. All the structural components are fabricated by 3D printing and the total weight of the robot is only ≈35 g.

In particular, the RFH‐module consists of one transmission leg and two driving components, as shown in Figure 2b. The transmission leg is designed to be rigid with an eccentric motor in the chamber, which is the key unit to excite the vibration. This rigid structure is facilitated to the effective and rapid conduction of vibration from the eccentric motor to the driving components, as well as the amplification of vibration amplitude. The two driving components are designed to be flexible: 1) A piece of thin rectangular polyvinyl chloride (PVC) plate is pasted above the rigid leg to form the flexible fin; this flexible fin can produce driving force on water surface more effectively for the deformation under vibration excitation. 2) The soft foot pasted under the leg is selected as a high elasticity ethylene‐vinyl acetate copolymer (EVA) foam with Shore hardness of 20 A, and it is cut into an L‐shape to reduce the structure stiffness; this soft foot avoids high‐frequency rigid contact with the ground, which can reduce the direct impact of vibration and weaken the damage to the RFH‐module. Furthermore, the elastic deformation of the soft foot can also serve as a buffer to adjust the contact and friction state with the ground to a certain extent autonomously, thus enhancing the stability and motion performance.

The two RFH‐modules are connected to the main body via soft silicone to avoid vibration coupling, which is also used to seal the connections. This soft connection can help to prevent vibration from being transmitted to the main body, thus, the two RFH‐modules can vibrate independently. In fact, this vibration isolation design is critical to ensure effective motion of the robot; otherwise, the robot would move randomly as the vibration coupling.

The internal part of the robot is the hardware of the control system, and it is mainly divided into three layers: the bottom layer is a rechargeable lithium battery for power supply, the middle layer is a DC motor driver and a Bluetooth low energy module, while the upper layer is a STM32 micro control unit.

In addition, as the flexible fin is supposed to vibrate on the water surface according to the aquatic locomotion mechanism, the water level line should be just below the lower surface of the flexible fin, which is achieved by adjusting the structure of the robot and has been illustrated in detail in Note S1, Supporting Information.

## 3 Terrestrial Locomotion Mechanism

The amphibious robot adopts the friction locomotion mechanism by the elliptical vibration of the soft foot for terrestrial motion, and it achieves the linear and rotational motions through the cooperation of two RFH‐modules.

**Figure 3:**
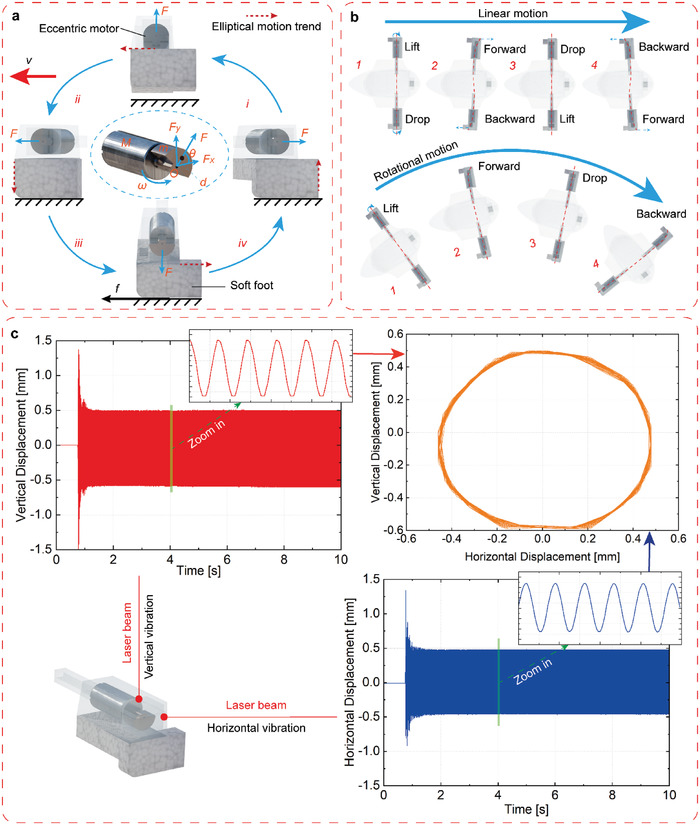
Friction locomotion mechanism for terrestrial motion. a) Frictional locomotion mechanism diagram of the RFH‐module in one cycle. b) Linear and rotational motion mechanisms of the robot in one cycle. c) The vibration trajectory of the soft foot.

The core unit of the RFH‐module is the eccentric motor, as shown in **Figure** **3**a. The motor will exert a centrifugal force when rotating, and the dynamic model can be obtained as:

(1) $$\left\{\begin{array}{c} F\left(t\right)=F_{0}e^{i\omega t}\\ F_{0}=\mathrm{md}\omega^{2} \end{array}\right.$$

where *F*(*t*) is the centrifugal force varied with time, *F*_0_ is the amplitude of the centrifugal force, *m* is the mass of the eccentric rotor, *d* is the eccentric distance, *ω* is the angular velocity. The direction of the centrifugal force is outward along the line connecting the mass center of eccentric rotor and the rotational center *O*. The horizontal and vertical components *F*_x_ and *F*_y_ of the centrifugal force are given as:

(2) $$\left\{\begin{array}{c} F_{x}\left(t\right)=F_{0}\cos\left(\omega t\right)\\ F_{y}\left(t\right)=F_{0}\sin\left(\omega t\right) \end{array}\right.$$

According to the dynamic model, the vibration strength has a square relationship with the angular velocity *ω*; and the angular velocity is positively related to the exerted voltage, as well as the vibration frequency *f* (see Figure S4b, Supporting Information). Therefore, the vibration amplitude is fully coupled with the frequency for the RFH‐module.

The RFH‐module will vibrate in elliptical trajectory under the centrifugal force generated by the eccentric motor. The inner end of the RFH‐module is softly connected on the main body, and the outer end vibrates in elliptical trajectory, so the whole RFH‐module exhibits a cone shape vibration (see Movie S1, Supporting Information). As shown in Figure 3a, the eccentric motor rotates counterclockwise, so that the soft foot exhibits a counterclockwise elliptical vibration. In the lower half of the elliptical trajectory, the rotor rotates into iii and iv regions, the soft foot is forced downward, and the maximum static frictional force increases. Meanwhile, the RFH‐module moves rightward relative to the ground, so the driving force to the left is obtained by the friction. In the upper half of the elliptical trajectory, the rotor rotates into i and ii regions, the RFH‐module will bounce up and leave the ground for the elasticity of the soft foot, and the frictional force decreases to zero, but the RFH‐module continues moving forward due to inertia. Thus, the ground will exert frictional force to the RFH‐module each time as the soft foot contacts with the ground in lower position. If the eccentric motor rotates reversely, the RFH‐module will exhibit a clockwise elliptical trajectory and move to the opposite direction.

The robot can realize various forms of motions on the ground through the cooperation of two RFH‐modules, as shown in Figure 3b. As for linear motion, the two RFH‐modules vibrate alternately with the same direction to achieve the motion of dual feet running. The rotational motion is realized via the velocity difference between the two RFH‐modules: if one RFH‐module keeps stationary and the other module vibrates elliptically, the robot can achieve rotation around the static foot to draw a circle, and it can achieve rotation in situ when the two RFH‐modules vibrate in reverse directions.

We design a specimen of the actual RFH‐module to measure the vibration trajectory at the end of the module using laser displacement sensors, as shown in Figure 3c. The motions in horizontal and vertical directions can be obtained separately, and the vibration states in two directions show nearly ideal sinusoidal vibrations by choosing a short time interval from the curve in stable zone. The combination of these two vibrations is an elliptical trajectory, which is in accordance with the desired motion.

## 4 Aquatic Locomotion Mechanism

The robot adopts the vibration‐induced flow mechanism to realize the aquatic locomotion. The fluid surface will be disturbed when objects vibrate on it, and specific shapes of waveform and flow fields will be generated. There are some relevant researches about the phenomena of flow fields generated by vibrating rigid blocks,^[^ ^31^, ^37^, ^38^ ^]^ while we utilize a thin plate as flexible fin to stimulate flow on water surface, which is more like a general case of rigid block with deformation. We also carry out relative studies of a rectangular block for comparison with the flexible plate in the same conditions, as illustrated in Note S4 and Figure S3, Supporting Information. It can be found that the thin flexible plate can produce water flow more effectively under the deformation of some specific vibration modes.

**Figure 4:**
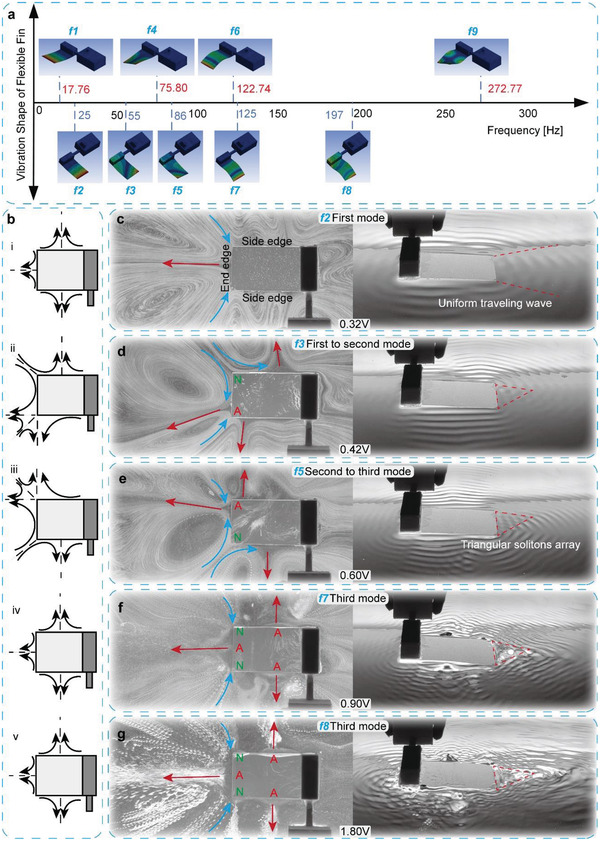
Water surface morphologies of the flexible plate under different frequencies. a) Deformations of the flexible plate under different frequencies. b) Flow filed variants diagram of the flexible plate. c) Water surface morphology at the first mode. d) Water surface morphology from the first to second mode. e) Water surface morphology from the second to third mode. f) Water surface morphology at the third mode. g) Water surface morphology at the third mode of higher voltage.

The flexible plate has a thin thickness and a low stiffness, resulting in obvious deformation when vibrating on water surface. Different vibration modes can be excited under different frequencies, which lead to complicated variations of surface flow fields. We first perform wet modal analysis and harmonious response analysis on the flexible plate to obtain vibration shapes under different frequencies, as shown in **Figure** **4**a. Compared to the vibration in air, the natural frequencies of the plate are significantly reduced for the adhesion of water. Since the deformations of the flexible plate at different vibration modes can be regarded as standing waves, we can assume that each point on the thin plate mainly vibrates in vertical direction according to the Kirchhoff‐Love Hypothesis; then the vertical displacement *z* of the flexible plate can be obtained as:

(3) z=Z(x,y)sin(ωt)

where *Z (x, y)* is the amplitude distribution function of the plate in vertical direction, *ω* is the vibration angular frequency.

Then the vertical velocity *v* of the plate can be derived as:

(4) v=ωZ(x,y)cos(ωt)

Thus, the velocity component in vertical direction of the fluid clusters in contact with the plate should be consistent with the vertical velocity of the plate, so the fluid dynamic pressure *q* is:

(5) $$q=\frac{1}{2}\rho {\left(\omega Z\left(x,y\right)\cos\left(\omega t\right)\right)}^{2}$$

where *ρ* is the density of fluid.

The fluid clusters in adhesion layer of the flexible plate are stimulated by the deformation to obtain different dynamic pressures. The fluid clusters near the antinodes of the edges get larger vibration velocities along vertical direction, and the fluid will be pushed both downward and outward. Considering the momentum equation and energy dissipation in incompressible viscous fluid, the antinodes will obtain lower pressures in the transverse direction of the fin, while the fluid clusters near the nodes have higher pressure likewise, which leads to a pressure gradient and fluid movement along the transverse direction. Thus, the fluid will flow from the nodes to the antinodes at the edges of the plate, and the variants of the theoretical flow fields under different vibration modes are represented in Figure 4b(i–v). A series of experiments, as shown in Movie S2, Supporting Information, are carried out to obtain the flow fields of a flexible plate on the water surface when different voltages are applied.

The first natural frequency of the flexible plate is 17.76 Hz according to the simulation (Figure 4a(f1)), and it corresponds to the voltage of 0.14 V. The free end of the plate flaps up and down at the bending mode with no torsion on the entire plate. In the experiment, a surface flow field similar to the vibrating rigid block is generated at the voltage of ≈0.30 V and the corresponding frequency of 35 Hz. There is an obvious outward flow (red line) in the middle of the end edge, and two backflows (blue line) appear at the corners forming two larger vortices with opposite rotational directions, as shown in Figure 4c. Uniformly propagating waves first appear on the surface during first mode process, and the wavefront is basically parallel to the edges of the plate. There is a deviation between simulation and experiment for the frequency, but this result is acceptable considering that the flow under low voltage is not perceptible as well as the errors during the experiment.

Asymmetrical deformations of the plate appear during the transitional state from the first to the second mode and also from the second to the third mode, due to the unilateral constraint and exerted force, as shown by the harmonic response simulation in Figure 4a(f3,f5). The deformations of the entire plate are inclined in two different directions for two transient states, and fluid clusters will flow from node (marked with green N) to antinode (marked with red A) due to the pressure gradient. Thus, these two different deformations produce unidirectional water flows in opposite directions along the end edge, with an inward flow under lower frequencies and an outward flow under higher frequencies, as shown in Figure 4d,e. As for surface wave, the wavenumber becomes larger and the waves become more intensive as voltage increasing, and finally, the whole surface wave breaks into several parts, and a triangular soliton array appears at the end edge due to the modulate instability.

For the third mode of the plate, the deformation at the middle of the end edge is larger than that of the two sides, as shown in the vibration shape of Figure 4a(f6). Therefore, the water flow will converge orientational from the nodes to the antinodes in middle at the end edge. In experiment, a distinct wave will gather and surge up at the outer side of the end edge first during the transition to the third mode (since flow gathers here at this stage, as shown in Figure 4e) and then gradually move toward the middle of the end edge, where the waves will merge together to form an undulating triangle wave area, as shown in Figure 4f. The fluid clusters gather at the middle and overcome the constraints of surface tension easily to produce an obvious outward flow. A pair of outward flows also appears on the two side edges of the fin for the same reason, but they are smaller compared with the end flow.

In summary, the flow field of the first mode is too weak as the low voltage and small vibration amplitude, and the flow fields around second mode are decentralized as transverse flows, while the third mode can gather water effectively in a wide frequency range at higher voltage. The fluid surface will be completely broken if increasing the voltage further, and water will be sprayed backward violently at the end edge of the flexible fin, thus producing a superior driving effect (see Figure 4g). Therefore, the third mode of the flexible fin can be adopted to achieve the aquatic motion by the vibration‐induced flow.

## 5 Motion Performance Experiments

### 5.1 Terrestrial Motion Performance

The robot can run on the ground with dual feet by alternated elliptical vibrations of the two RFH‐modules. We mainly evaluate the linear speed, rotational speed, and obstacle crossing ability for terrestrial motion.

**Figure 5:**
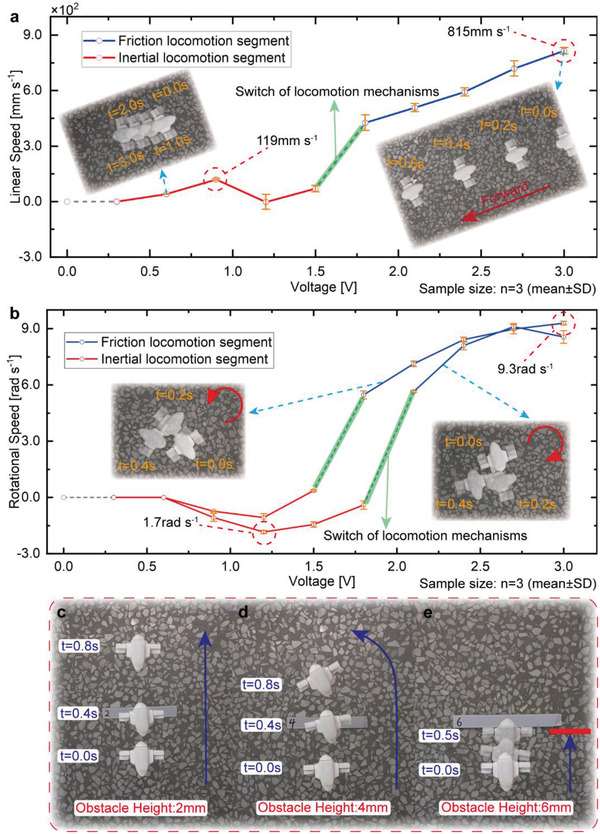
Terrestrial motion performances and obstacle crossing ability. a) The variation of the linear speed with the exciting voltage. b) The variation of the rotational speed with the exciting voltage. c) Crossing obstacle of 2 mm. d) Crossing obstacle of 4 mm. e) Crossing obstacle of 6 mm.

The robot realizes linear motion by exerting Pulse Width Modulation (PWM)  adjusted voltage on the two RFH‐modules. The linear speed of the robot versus voltage, as well as the motion trajectories on the ground, can be obtained by the frame superposition method in a series of motion movies. As shown in **Figure** **5**a, the maximum linear speed of the robot can reach 815 mm s^–1^ at the voltage of 3.0 V, and the relative speed is ≈10.9 Body Length s^–1^ (BL s^–1^). The friction locomotion segment at high voltage exhibits good linearity, and it decreases as the voltage decreases. However, there is an interesting phenomenon when the voltage is lower. The speed will decrease and approach to zero rapidly as the voltage is reduced to ≈1.5 V, the robot can only perform back and forth motion in situ although the eccentric motors still work steadily(see Movie S3, Supporting Information). In this case, the RFH‐module is in the switching state between the friction locomotion mechanism and inertial locomotion mechanism, which can generate opposite motion directions (see Note S3, Supporting Information). The robot adopts the inertial locomotion mechanism when the voltage is further reduced, and it can run forward again and reach a maximum speed of 119 mm s^–1^ at 0.9 V. Then, the speed drops to zero again when the voltage is too small to overcome the friction.

The robot achieves rotational motion by setting one RFH‐module still and exerting voltage on the other one (see Movie S4, Supporting Information). The relationship between the voltage and rotational speed is tested, see Figure 5b. The maximum rotational speed can reach 9.2 rad s^–1^ at the voltage of 3.0 V. The trend of rotational speed curve is basically consistent with the linear speed and shows linearity in friction locomotion segment. However, there is a little deviation between the clockwise and counterclockwise rotational speed curves due to the differences of the two RFH‐modules. Similar phenomenon as in linear motion experiments appears when the voltage gradually decreases. The rotational speed quickly drops to zero as the robot reaches the switching state of two locomotion mechanisms at ≈1.5 V and cannot produce effective motion; then, the robot switches to the inertial locomotion mechanism when the voltage is further reduced, and it rotates reversely without changing the rotational direction of motor. The reverse rotational speed can reach to a peak of ≈1.7 rad s^–1^. It should be noted that this reverse motion is expected motion motivated by inertial mechanism according to the analysis in Note S3, Supporting Information, which is different from the linear motion. The reason for the anomalous linear motion may be that the coupling of vibrations on two sides affects the overall vibration state.

As for obstacle crossing ability, we set obstacles with an increasing gradient of heights on the ground until the robot cannot pass it (Figure 5c–e). The robot can easily surpass obstacles with a height of 2 mm without affecting its own motion state. It can cross obstacles up to 4 mm in height, which is around one fifth of the robot height. In this case, one of its feet usually crosses first, then the other foot passes, which will change the movement direction after passing the obstacle. However, for obstacles with a height of 6 mm and above, the robot will jump repeatedly in front of the obstacle and cannot pass. The obstacle crossing movies are shown in Movie S6, Supporting Information. In addition, we test the motion performances of the robot on different substrates, which are glass, leather, wood, foam, carpet, and sand, respectively. The robot shows satisfying speed on these surfaces with different roughness and flatness. The terrestrial motion movies of the robot on different substrates are shown in Movie S8, Supporting Information.

### 5.2 Aquatic Motion Performance

The robot utilizes two flexible fins, which vibrate in a desired frequency range to excite the third mode shown in Figure 4, to realize the aquatic locomotion; and it can achieve the linear motion and rotational motion on the water surface via the cooperation of these two RFH‐modules(see Movie S5, Supporting Information).

The vibration frequency of the RFH‐module is controlled by the exciting voltage applied to the eccentric motor, and different vibration modes can be excited on the flexible fin under different frequencies. According to the aquatic locomotion mechanism, the locomotion effect occurs at the third mode of the flexible fin, and the natural frequency is ≈120 Hz, corresponding to the voltage of ≈1.0 V. Then, there will be a wide frequency range to achieve the effective locomotion where the deformation of the flexible fin is similar to the third mode, as shown in the harmonic simulation in Figure 4.

**Figure 6:**
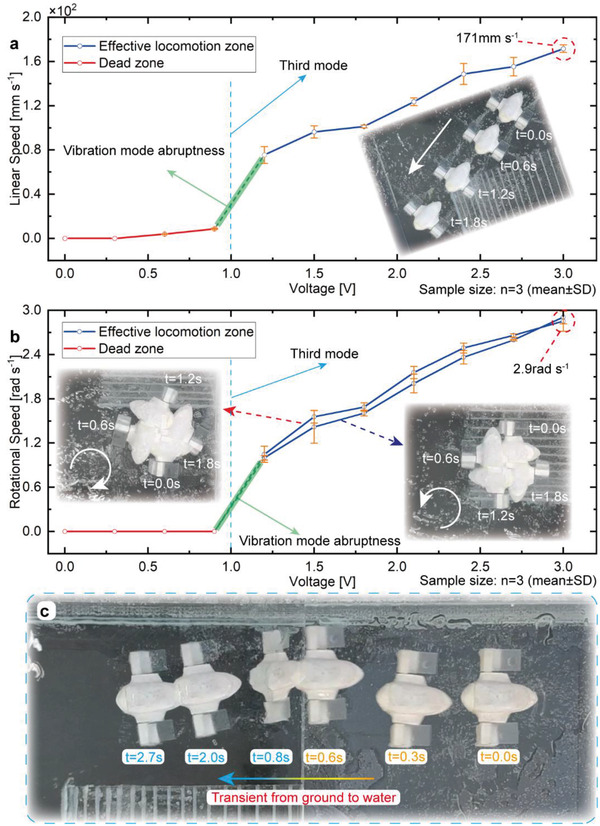
Aquatic motion performances and amphibious switching motion. a) The variation of the linear speed on water surface with the exciting voltage. b) The variation of the rotational speed on water surface with the exciting voltage. c) The amphibious switching motion when dropping into water from ground.

We test the linear speeds under different voltages by the frame superposition method first, and the curve of the linear speed with voltage is shown in **Figure** **6**a. The swimming speed shows a relatively stable linear increasing trend in the effective locomotion zone as the voltage increases. The maximum swimming speed is ≈171 mm s^–1^ at 3.0 V with the corresponding frequency of 230 Hz, and the relative swimming speed reaches ≈2.3 BL s^–1^. The swimming speed will enter the dead zone when the voltage is reduced to ≈0.9 V; and the corresponding vibration frequency of the module is ≈110 Hz, which is just the frequency out of the effective frequency range; thus, the flexible fin cannot produce effective driving force. This phenomenon is consistent well with the theoretical analysis of aquatic locomotion mechanism.

The relationship between the rotational speed and the voltage is measured by similar method (Figure 6b). The rotational speed curves also have effective locomotion zones and dead zones. The maximum rotational speed on the water surface is ≈2.9 rad s^–1^, and it decreases steadily as the voltage decreases in the effective locomotion zone; then, it drops significantly and approaches to zero when the voltage is reduced to ≈0.9 V. In addition, although the robot is designed to work on the water surface theoretically, it can still generate driving force to some extent when its flexible fins are submerged near the surface in practice. This ability guarantees that the robot can even maintain swimming motion when it is overturned, which demonstrates the good resistance to harsh aquatic conditions (also see Movie S6, Supporting Information).

The amphibious motion when the robot drops into water is tested in a special designed water tank fabricated by glass (Figure 6c). The experiments show that the best terrestrial and aquatic motion performances both appear at the voltage of 3.0 V. Therefore, the matched locomotion parameters guarantee that the robot can enter water from ground directly and realize the conversion of locomotion modes efficiently. In addition, we also test the amphibious switching characteristics of the robot, and the experiments show that the robot is able to switch between the two environments through a slope successfully (also see Movie S6, Supporting Information).

## 6 Conclusion and Discussion

A miniature amphibious robot is proposed based on the vibration‐driven locomotion mechanism. We design an RFH‐module as the core locomotion component for realizing the amphibious motions. The robot adopts friction locomotion mechanism with dual feet for terrestrial motion and the phenomena caused by inertial locomotion mechanism are also revealed. We also elucidate the influence of vibration modes on the morphologies of the surface flow field theoretically and experimentally. The results prove that the third mode of the flexible fin produces the best locomotion effect, which provides a new design idea for aquatic motion.

For the terrestrial motion, it should be noted that the existence of inertial locomotion mechanism makes it a bit complicated as illustrated in above experiment. The friction locomotion mechanism will fail when the voltage on motor is too small to excite the elliptical vibration, but the robot can still move by centrifugal inertial mechanism. The moving directions of these two mechanisms are opposite when the rotational direction of the motor keeps the same, so that effective combination of these two locomotion mechanisms may provide a more flexible terrestrial locomotion scheme.

For the aquatic locomotion mechanism, the outward flow is first stimulated during the experiment of the rigid vibrating block; however, the flow direction turns into inward suddenly when the voltage is high (see Figure S3, Supporting Information). As for flexible plate, the highest frequency that the eccentric motor can excite has been reached in the experiment, but there is no inward flow compared with rigid block. The reason may be that the deformations at free edges of the flexible fin promote the convergence of water flow, which is not conducive to the generation of stable standing wave. In addition, the deformation of the flexible fin is not a simple vertical displacement, but an oblique flip. This oblique vibration can produce a horizontal component to push the water outward, which enhances the ability of water to flow outward and further ensures the effective locomotion on water.

**Figure 7:**
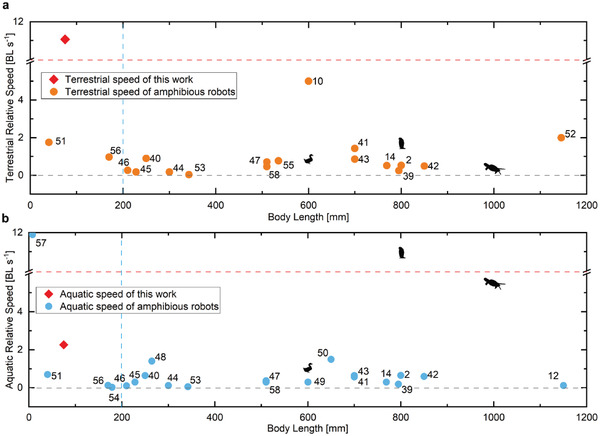
Performance comparison of the proposed amphibious robot with some other robots and animals. a) Comparisons of the terrestrial relative speeds. b) Comparisons of the aquatic relative speeds.

The robot achieves miniaturization with no transmission mechanism or joints, and its size is smaller than most existing amphibious robots, as shown in **Figure** **7**, even though the power supply and the whole control system are integrated. This robot exhibits superior performance on relative motion speed compared with other typical amphibious robots.^[^ ^2^, ^10^, ^12^, ^14^, ^39^, ^40^, ^41^, ^42^, ^43^, ^44^, ^45^, ^46^, ^47^, ^48^, ^49^, ^50^, ^51^, ^52^, ^53^, ^54^, ^55^, ^56^, ^57^, ^58^ ^]^ In particular, the relative terrestrial speed reaches to ≈10.9 BL s^–1^, which shows the significant superiority of this design. The relative aquatic speed is ≈2.3 BL s^–1^, which is not that fast as the terrestrial speed, but still better than most amphibious robots. It is worth noting that some robots are excited by external magnetic field,^[^ ^18^, ^57^ ^]^ which facilitates their miniaturization. However, the excitation source is not possible to be integrated. They belong to unconventional amphibious robots with different objectives, so they are less comparative.

The amphibious microrobot has unique advantages of high flexibility and high concealment in the complex terrestrial and aquatic environmental conditions, due to its small size and strong environmental adaptability. By combining with intelligent control methods, it will realize autonomous obstacle avoidance, trajectory planning, and attitude adjustment in future practical applications. Meanwhile, multiple robots can build up a robot cluster to realize cooperative motions and perform tasks. They can freely switch between terrestrial and aquatic environments, enter small caves and pass through narrow amphibious passages to achieve the objectives of information acquisition, disaster relief, and resource detection.

There are still some imperfections in this work, for example, the endurance of the robot is restricted to a working time of 25 min due to the limitation of the power supply capacity; the power supply, occupying about half size of the control system, also limits the miniaturization of the overall size and the integration of other control elements. The emergence of more efficient and smaller power supply in the future may help to miniaturize the robot further.

## 7 Experimental Section

### Materials and Fabrication of Robot

All the structural components of the amphibious robot were fabricated by 3D printing, which reduced the cost and made it possible for batch production, thus it was convenient to achieve further research such as the performance improvements and the collaboration of robot cluster. The 3D printing material was chosen as polylactic acid with fused deposition modeling method, and the mass could be adjusted by the filling percent of 3D printing to ensure that the lower surface of fin exactly floated on water surface. The EVA foam was selected as the soft foot material for its good elasticity, lightweight, and lower water absorption. The linear elastic limit *σ*_e_ of the EVA foam was ≈0.1–0.2 MPa under uniaxial compression; thus, the soft foot was in the elastic deformation range during the vibration process. The material of the flexible fin was selected as frosted PVC plate, whose stiffness was moderate to ensure the desired deformation under the excitation of eccentric motor, and it could also avoid uncontrollable and large passive deformation due to the influence of water flow.

### Vibration Trajectory Measurement of RFH‐Module

The vibration trajectory of the RFH‐module was measured by two laser displacement sensors (Keyence LK‐H020, Japan), in which the maximum sampling frequency was 392 kHz and dynamic resolution was 5 nm. The sampling frequency was chosen as 5 kHz, which was ≈25 times the maximum frequency of the eccentric motor. Two orthogonally placed laser displacement sensors collected signals at the end of the RFH‐module simultaneously to get the vibration displacement in horizontal and vertical directions, respectively, and then the obtained signals were synthesized further to obtain the vibration trajectory.

### Experiment Design of Aquatic Locomotion Mechanism

This experiment aimed to investigate and verify the influence of vibration modes of the flexible plate on the surface flow field. One rigid and one flexible specimen were designed respectively to perform the experiments, as shown in Figure S6, Supporting Information. The specimens were powered by a DC regulated power supply, so that the amplitude and frequency could be adjusted by adjusting the voltage. The clamping device utilized a gimbaled magnetic base with a three‐jaw clamp, and the bottom of the specimen just contacted the fluid surface by adjusting the height of the magnetic base. A simplified particle image velocimetry method was used to observe the path line of the flow field. All the devices were placed in a dark environment with a LED emitting tube placed at the side. The PE powder with diameter of ≈50 µm was used as tracer particles to observe the traces on fluid surface, whose density was slightly lower than that of water and insoluble in water. Dispersant was added to ensure that the powder could distribute uniformly on the water surface, and then the particle trajectory on the surface in steady state could be obtained by recording through camera and superimposing frames.

### Motion Performances Measurement

Due to the fast movement speed and wide motion range for terrestial motion, the frame superposition method was adopted to obtain the motion trajectories from motion movies and calculate the speed. During the measurement of linear speed, the eccentric motors were calibrated in advance in case of the differences in characteristics between motors, and the exerted voltage on each motor was adjusted by PWM signal. The maximum voltage was applied first and then the voltage was gradually reduced to obtain a series of motion movies and calculate the movement speed under different voltages. During the measurementof rotational speed, the voltage on one RFH‐module was kept zero, and the voltage on the other RFH‐module was gradually reduced to avoid the influence of locomotion mechanism switching. In this case, the robot rotated with the static driving foot as center and the total width of the robot as radius to draw a circle. While dealing with the motion movies, one point on the robot was chosen as the marker to obtain the motion trajectories, and several movement durations were selected separately to calculate an average speed. As for the rotational motion, several markers on the robot were fitted to a circle to obtain the rotational center and calculate the rotational speed. For the experiment of crossing obstacles, different layers of tapes were pasted on the ground to obtain a gradient increasing obstacle height, and the height unable to overrun prevailed. As for the aquatic motion, the basic method was the same as the terrestrial motion experiment except that the measurement was carried out in a specially designed water tank. The motion movies at different voltages were shot and analyzed subsequently by frame to obtain the motion trajectories and the speeds.

### Statistical Analysis

The sample size (*n*) for each statistical analysis was *n* = 3. The data were expressed as mean ± SD (Standard Deviation). Statistical analysis of the data was performed using OriginPro 2021b.

## Conflict of Interest

The authors declare no conflict of interest.

## Supporting information

Supporting Information

Supplemental Movie 1

Supplemental Movie 2

Supplemental Movie 3

Supplemental Movie 4

Supplemental Movie 5

Supplemental Movie 6

Supplemental Movie 7

Supplemental Movie 8

## Data Availability

The data that support the findings of this study are available from the corresponding author upon reasonable request.

## References

[1] J. Delmerico , S. Mintchev , A. Giusti , B. Gromov , K. Melo , T. Horvat , C. Cadena , M. Hutter , A. Ijspeert , D. Floreano , L. M. Gambardella , R. Siegwart , D. Scaramuzza , J. Field Rob. 2019, 36, 1171.

[2] L. Cui , P. Cheong , R. Adams , T. Johnson , J. Mech. Des. 2014, 136, 115001.

[3] G. Freitas , G. Gleizer , F. Lizarralde , L. Hsu , N. R. S. dos Reis , J. Field Rob. 2010, 27, 197.

[4] G. Picardi , M. Chellapurath , S. Iacoponi , S. Stefanni , C. Laschi , M. Calisti , Sci. Rob. 2020, 5, eaaz1012. 10.1126/scirobotics.aaz1012 33022623

[5] B. B. Dey , S. Manjanna , G. Dudek , in 2013 IEEE/RSJ International Conference on Intelligent Robots and Systems, IEEE, Piscataway, NJ 2013, pp. 5622–5628.

[6] R. Baines , S. Freeman , F. Fish , R. Kramer‐Bottiglio , Bioinspir. Biomim. 2020, 15, 025002. 31914424 10.1088/1748-3190/ab68e8

[7] J. Yu , R. Ding , Q. Yang , M. Tan , J. Zhang , J. Field Rob. 2013, 30, 702.

[8] K. Tadakuma , R. Tadakuma , M. Aigo , M. Shimojo , M. Higashimori , M. Kaneko , in 2011 IEEE International Conference on Robotics and Automation, IEEE, Piscataway, NJ 2011, pp. 3523–3524.

[9] S. Guo , Y. He , L. Shi , S. Pan , R. Xiao , K. Tang , P. Guo , Rob. Comput.‐Integr. Manuf. 2018, 51, 37.

[10] V. Kaznov , M. Seeman , in 2010 IEEE/RSJ International Conference on Intelligent Robots and Systems, IEEE, Piscataway, NJ 2010, pp. 5113–5118.

[11] J. G. Marquardt , J. Alvarez , K. D. von Ellenrieder , IEEE J. Oceanic Eng. 2014, 39, 641.

[12] N. Li , S. Ma , M. Wang , B. Li , Y. Wang , in 2012 IEEE/RSJ International Conference on Intelligent Robots and Systems, IEEE, Piscataway, NJ 2012, pp. 2282–2288.

[13] A. J. Ijspeert , A. Crespi , D. Ryczko , J.‐M. Cabelguen , Science 2007, 315, 1416. 17347441 10.1126/science.1138353

[14] A. Crespi , A. J. Ijspeert , IEEE Trans. Rob. 2008, 24, 75.

[15] J. Hwang , W. D. Wang , Adv. Mater. Technol. 2022, 7, 2101153.

[16] C. Xu , Z. Yang , S. W. K. Tan , J. Li , G. Z. Lum , Adv. Intell. Syst. 2022, 4, 2100259.

[17] K. Ren , J. Yu , Ocean Eng. 2021, 227, 108862.

[18] W. Hu , G. Z. Lum , M. Mastrangeli , M. Sitti , Nature 2018, 554, 81. 29364873 10.1038/nature25443

[19] X. Du , H. Cui , T. Xu , C. Huang , Y. Wang , Q. Zhao , Y. Xu , X. Wu , Adv. Funct. Mater. 2020, 30, 1909202.

[20] J. Zhang , Y. Guo , W. Hu , R. H. Soon , Z. S. Davidson , M. Sitti , Adv. Mater. 2021, 33, 2006191.

[21] Y. Wu , J. K. Yim , J. Liang , Z. Shao , M. Qi , J. Zhong , Z. Luo , X. Yan , M. Zhang , X. Wang , R. S. Fearing , R. J. Full , L. Lin , Sci. Rob. 2019, 4, eaax1594. 10.1126/scirobotics.aax1594 33137774

[22] Y. Liu , J. Li , J. Deng , S. Zhang , W. Chen , H. Xie , J. Zhao , Adv. Intell. Syst. 2021, 3, 2100015.

[23] Z. Ren , S. Kim , X. Ji , W. Zhu , F. Niroui , J. Kong , Y. Chen , Adv. Mater. 2022, 34, 2106757. 10.1002/adma.202106757 34839551

[24] C. Tang , W. Ma , B. Li , M. Jin , H. Chen , Adv. Eng. Mater. 2020, 22, 1901130.

[25] P. Vartholomeos , K. Vlachos , E. Papadopoulos , IEEE Trans. Autom. Sci. Eng. 2013, 10, 545.

[26] Q. Su , S. Zhang , Y. Liu , J. Deng , IEEE Trans. Ind. Electron. 2021, 68, 1466.

[27] K. Nagaoka , K. Yoshida , in 2013 IEEE/RSJ International Conference on Intelligent Robots and Systems, IEEE, Piscataway, NJ 2013, pp. 763–768.

[28] S. Iyobe , M. Shimizu , T. Umedachi , IEEE Rob. Autom. Lett. 2022, 7, 992.

[29] Z. Li , N. V. Myung , Y. Yin , Sci. Rob. 2021, 6, eabi4523. 10.1126/scirobotics.abi4523 34851711

[30] D. Jiang , Z. Fan , H. Wang , M. Xu , G. Chen , Y. Song , Z. L. Wang , ACS Nano 2020, 14, 15394. 33179494 10.1021/acsnano.0c05901

[31] H. Punzmann , N. Francois , H. Xia , G. Falkovich , M. Shats , Nat. Phys. 2014, 10, 658.

[32] M. Wiklund , R. Green , M. Ohlin , Lab Chip 2012, 12, 2438. 22688253 10.1039/c2lc40203c

[33] T. Qiu , S. Palagi , A. G. Mark , K. Melde , F. Adams , P. Fischer , Adv. Mater. Interfaces 2017, 4, 1700933. 10.1021/acsami.7b12755 PMC5730945 29148713

[34] M. Kaynak , P. Dirix , M. S. Sakar , Adv. Sci. 2020, 7, 2001120. 10.1002/advs.202001120 PMC7578873 33101852

[35] N. Francois , H. Xia , H. Punzmann , M. Shats , Phys. Rev. Lett. 2013, 110, 194501. 23705709 10.1103/PhysRevLett.110.194501

[36] C. Roh , M. Gharib , Proc. Natl. Acad. Sci. USA 2019, 116, 24446. 31740588 10.1073/pnas.1908857116 PMC6900504

[37] S. Taneda , J. Fluid Mech. 1991, 227, 193.

[38] F. Moisy , G.‐J. Michon , M. Rabaud , E. Sultan , Phys. Fluids 2012, 24, 022110.

[39] S. Zhang , Y. Zhou , M. Xu , X. Liang , J. Liu , J. Yang , IEEE/ASME Trans. Mechatron. 2016, 21, 1720.

[40] M. Li , S. Guo , H. Hirata , H. Ishihara , Rob. Auton. Syst. 2015, 64, 21.

[41] Y. Yi , Z. Geng , Z. Jianqing , C. Siyuan , F. Mengyin , in 2015 IEEE/RSJ International Conference on Intelligent Robots and Systems (IROS), IEEE, Piscataway, NJ 2015, pp. 559–566.

[42] A. Crespi , K. Karakasiliotis , A. Guignard , A. J. Ijspeert , IEEE Trans. Rob. 2013, 29, 308.

[43] J. Yu , R. Ding , Q. Yang , M. Tan , W. Wang , J. Zhang , IEEE/ASME Trans. Mechatron. 2012, 17, 847.

[44] H. Xing , S. Guo , L. Shi , X. Hou , Y. Liu , H. Liu , Y. Hu , D. Xia , Z. Li , in 2019 IEEE/RSJ International Conference on Intelligent Robots and Systems (IROS), IEEE, Piscataway, NJ 2019, pp. 1702–1707.

[45] H. Jin , E. Dong , G. Alici , S. Mao , X. Min , C. Liu , K. H. Low , J. Yang , Bioinspir. Biomim. 2016, 11, 056012. 27609700 10.1088/1748-3190/11/5/056012

[46] A. A. M. Faudzi , M. R. M. Razif , G. Endo , H. Nabae , K. Suzumori , in 2017 IEEE International Conference on Advanced Intelligent Mechatronics (AIM), IEEE, Piscataway, NJ 2017, pp. 981–986.

[47] B. Zhong , S. Zhang , M. Xu , Y. Zhou , T. Fang , W. Li , IEEE/ASME Trans. Mechatron. 2018, 23, 542.

[48] A. Crespi , D. Lachat , A. Pasquier , A. J. Ijspeert , Auton. Rob. 2008, 25, 3.

[49] M. Cianchetti , M. Calisti , L. Margheri , M. Kuba , C. Laschi , Bioinspir. Biomim. 2015, 10, 035003. 25970014 10.1088/1748-3190/10/3/035003

[50] C. Georgiades , A. German , A. Hogue , H. Liu , C. Prahacs , A. Ripsman , R. Sim , L.‐A. Torres , P. Zhang , M. Buehler , G. Dudek , M. Jenkin , E. Milios , in 2004 IEEE/RSJ International Conference on Intelligent Robots and Systems (IROS), IEEE, Piscataway, NJ 2004, pp. 3525–3531.

[51] Y. Chen , N. Doshi , B. Goldberg , H. Wang , R. J. Wood , Nat. Commun. 2018, 9, 2495. 29950597 10.1038/s41467-018-04855-9 PMC6021446

[52] M. A. Klein , A. S. Boxerbaum , R. D. Quinn , R. Harkins , R. Vaidyanathan , in 2012 4th IEEE RAS & EMBS International Conference on Biomedical Robotics and Biomechatronics (BioRob), IEEE, Piscataway, NJ 2012, pp. 1335–1340.

[53] E. Milana , B. V. Raemdonck , K. Cornelis , E. Dehaerne , J. D. Clerck , Y. D. Groof , T. D. Vil , B. Gorissen , D. Reynaerts , in 2020 3rd IEEE International Conference on Soft Robotics (RoboSoft), IEEE, Piscataway, NJ 2020, pp. 766–771.

[54] Y. Tang , Q. Zhang , G. Lin , J. Yin , Soft Rob. 2018, 5, 592. 10.1089/soro.2017.0133 29957129

[55] G. Wang , X. Chen , S. Yang , P. Jia , X. Yan , J. Xie , Mechatronics 2017, 48, 1.

[56] M. Wu , X. Xu , Q. Zhao , W. H. Afridi , N. Hou , R. H. Afridi , X. Zheng , C. Wang , G. Xie , Adv. Mater. Technol. 2022, 2200536.

[57] Q. Ze , S. Wu , J. Dai , S. Leanza , G. Ikeda , P. C. Yang , G. Iaccarino , R. R. Zhao , Nat. Commun. 2022, 13, 3118. 35701405 10.1038/s41467-022-30802-w PMC9198078

[58] J. Zhu , T. Fang , M. Xu , Y. Zhou , W. Huang , S. Zhang , in 2018 IEEE International Conference on Real‐Time Computing and Robotics (RCAR), IEEE, Piscataway, NJ 2018, pp. 417–420.

