# Supplementary material for: Miniature Amphibious Robot Actuated by Rigid‐Flexible Hybrid Vibration Modules
Source: Adv Sci (Weinh). 2022 Aug 18;9(29):2203054. doi: 10.1002/advs.202203054 (PMC9561757; doi:10.1002/advs.202203054)
Supplement: Supplementary file 1 — Supporting Information [file ADVS-9-2203054-s007.pdf]

ADVANCED SCIENCE
Open Access

Supporting Information

for *Adv. Sci.*, DOI 10.1002/advs.202203054

Miniature Amphibious Robot Actuated by Rigid-Flexible Hybrid Vibration Modules

Dehong Wang, Yingxiang Liu, Jie Deng, Shijing Zhang, Jing Li, Weiyi Wang, Junkao Liu, Weishan Chen, Qiquan Quan, Gangfeng Liu, Hui Xie and Jie Zhao**

Supporting Information

Miniature Amphibious Robot Actuated by Rigid-flexible Hybrid Vibration Modules

Dehong Wang, Yingxiang Liu, Jie Deng, Shijing Zhang, Jing Li, Weiyi Wang, Junkao Liu, Weishan Chen, Qiquan Quan, Gangfeng Liu, Hui Xie, Jie Zhao**

*Corresponding author. Email: liuyingxiang868@hit.edu.cn (Y.L.); jzhao@hit.edu.cn (J.Z.)

Note S1. Configuration modification for buoyancy balance and stability analysis

The configuration of the robot needs to be modified to ensure best locomotion performance. According to aquatic locomotion mechanism, the flexible fins are supposed to vibrate on the water surface, so that the water level should be just below the lower surface of the fin. The water level line is the contact trajectory between the robot and the water, and it is mainly determined by the density and shape of the robot. The buoyancy on the robot is equal to the weight of discharged fluid, and the direction is vertical upward from Archimedes principle. Therefore, the overall mass of the robot should be equal to the mass of the discharged water below the fin.

This amphibious robot mainly includes the internal electronic components of the control system and the external structural components, as shown in Figure 2. The size and mass parameters of each electronic component are listed in Table S1, while the materials of the structural components can be adjusted for the desired buoyancy balance. We first establish the whole model in SOLIDWORKS and evaluate the volume below the fin to obtain the buoyancy force, and then the water level can be adjusted to the desired position by adjusting the density of the structural components, as shown in Figure S1a. Although the mass of the actual prototype may have some deviation from the model, the size of the soft foot can be fine-tuned to ensure the water level.

There will be certain changes of water density when the robot moves in different water conditions. The simulation shows that the water level only drops 0.38 mm as the robot enters from freshwater environment (ρ = 1000 kg m^{-3}) to seawater environment (ρ = 1025 kg m^{-3}). This change hardly affects the actual movement on the water surface, which indicates that the robot can obtain good locomotion capability spanning different water environments.

Moreover, the motion stability is also an important issue to be considered. For motion on water surface, the stability is related to the relative position of the gravity center and buoyancy center and the robot's external shape. This robot is designed as a relative flat shape, it adopts the configuration of tight at bottom and wider at top, which can lift the buoyancy center; further, the density distribution is adjusted to increase the mass of the lower part, so as to lower the gravity center. The main method to estimate the stability is through metacenter, which is the curvature center of the buoyancy curve; the robot is stable if the gravity center is lower than the metacenter; otherwise, unstable. Both transverse stability and lateral stability should be analyzed for this robot, as there is no obvious size difference in the two directions. As shown in Figure

S1b, the buoyancy centers at different angles can be obtained, and then the metacenter can be acquired according to the definition of metacenter. The positions of the metacenters can be obtained separately when the robot is tilted forward by 5°, backward by 5°, and lateral by 5°, and these positions are higher than the positions of the gravity centers, which means that the robot is stable when moving on the water surface.

Note S2. Resistance analysis for aquatic motion

The resistances on the robot need to be considered when moving in water, which mainly includes viscous resistance and pressure resistance. The viscous resistance mainly depends on the surface roughness; while the pressure resistance (includes viscous pressure resistance and wave-making resistance), mainly relates to the shape of object. As shown in Figure S1c, we perform multiphase simulation by adopting the VOF multiphase model and the SST k-ω turbulence model. The free surface is set at the flexible fin with the upper part as air and the lower part as water. The simulation shows that the total resistance is about 0.025 N, in which the viscous resistance is about 0.0016 N, only accounting for 6.4% of the total resistance; so, the resistance is dominated by the pressure resistance. The streamline shows that the water flow fits well on the underneath surface in front part, but the boundary layer separates at the end corners resulting in vortex, which generates pressure resistance. Although the boundary layer separation cannot be completely avoided, the streamline design reduces the resistance to a certain extent. In addition, the wave making resistance is also not negligible. As shown in Figure S1c, the front of the robot will encounter water leading to surging up of surface and excite a set of head waves and tail waves. But this robot utilizes the surface flow to locomotion, so it is difficult to judge the role of the wave-making resistance during the motion of the robot.

Note S3. Inertial locomotion mechanism

The inertial locomotion mechanism is realized by the periodic motion of inertial mass and the asymmetric resistance with the ground. The driving force is the centrifugal force generated by the eccentric motor. As shown in Figure S2a, a plane model can be obtained by simplifying the actual RFH-module. The eccentric motor is enclosed in a rigid shell and an elastic foot is pasted at the bottom. The rotor is naturally downwards initially and rotates counterclockwise, and the rotation angle of the eccentric rotor is θ. We build this model in ADAMS and perform simulation by setting the rotary speed as 350 rad s^{-1} and the static and dynamic friction coefficient as 0.5 and 0.4, respectively. The motion curves of the module can be obtained separately (Figure S2b-

d). As shown in the curves, one motion cycle can be divided into 4 stages. 1) Stage 1, the eccentric rotor stays in region iv, and the rotational angle is less than the critical value for the horizontal component of the centrifugal force (F_x) to overcome the static friction, so the module remains stationary. 2) Stage 2, the acceleration suddenly increases as F_x overcomes the friction, and the robot begins to accelerate in positive direction; the acceleration starts to decrease to zero when the rotor reaches to latter part of region i, while the velocity continues increasing. 3) Stage 3, the module starts decelerating, it will decelerate under both frictional and centrifugal force when the rotor enters region ii, and the velocity gradually decelerates to zero. 4) Stage 4, the module begins to move reversely, and the direction of the friction changes, thus the acceleration changes abruptly. After a short stage of reverse motion, the velocity decelerates to zero again.

In one period, the eccentric rotor is in the upper half of the elliptical trajectory when the robot moves forward, so that the frictional force is reduced and the acceleration is higher, but the deceleration is lower. The eccentric rotor is in the lower half of the elliptical trajectory when the robot moves backwards resulting in greater friction, so that the acceleration in reverse direction is lower, but the deceleration is higher. Due to the difference of friction in each period, the module shows more forward motion than backward motion, so that unidirectional motion appears.

The vertical displacement can be negligible due to the small driving force at low voltage. Ignoring the influence of the elastic deformation of the driving foot, the force equations in two directions can be obtained as:

$$\begin{cases} (M+m)a_x = F_x + f \\ (M+m)g = N + F_y \end{cases} \tag{1}$$

where m is the mass of the eccentric rotor, M is the mass of the whole RFH-module except the eccentric motor and the elastic foot, a_x is the acceleration of the module in horizontal direction, f is the horizontal frictional force between the module and the ground, F_x is the horizontal component of the centrifugal force, F_y is the vertical component of the centrifugal force, and N is the support force of the ground. In the vertical direction, the RFH-module keeps in the static equilibrium state, so that the direction of the frictional force varies with this regularity in one period:

$$f=\begin{cases}-\mu_{\mathrm{d}} N \operatorname{sgn}\left(v_{\mathrm{x}}\right) & v_{\mathrm{x}} \neq 0 \\ -F_{\mathrm{x}} \operatorname{sgn}\left(F_{\mathrm{x}}\right) & v_{\mathrm{x}}=0, a_{\mathrm{x}}=0 \\ -\mu_{\mathrm{s}} N \operatorname{sgn}\left(F_{\mathrm{x}}\right) & v_{\mathrm{x}}=0, a_{\mathrm{x}} \neq 0\end{cases} \tag{2}$$

where sgn is the Sign function, μ_{d} is the dynamic coefficient of friction, μ_{s} is the static coefficient of friction, v_{x} is the velocity in horizontal direction. The three conditions in Equation S2 represent three motion states, no motion and no motion trend state, and no motion but about to move state separately. The motion curve of the RFH-module calculated by this model is basically the same as the simulation result.

It is worth noting that when the rotational direction of the motor for inertial locomotion mechanism is the same as the friction locomotion mechanism, the generated movements are in opposite directions. Thus, the frictional driving force is equivalent to the centrifugal driving force during the switching process between two locomotion mechanisms, and the driving force is correspondingly cancelled, so that there will be a state of irregular jumping in situ. This can explain the phenomenon that the robot stays in still when the voltage is reduced to around 1.5 V but moves reversely as the voltage is further reduced.

The switching condition occurs when the elastic foot starts to detach from the ground. A forced vibration system in vertical direction can be obtained by decomposing the centrifugal force of the rotor (Figure S2e). The dynamic equation in the vertical direction can be obtained directly by the D'Alembert's principle as:

$$(M-m)\ddot{y}+m\frac{d^2}{dt^2}(y+d\sin(\omega t))+c\dot{y}+ky=0 \tag{3}$$

where y is the displacement in vertical direction, d is the eccentric distance, ω is the angular velocity of the eccentric rotor, c is the equivalent viscous damping coefficient of the soft foot, k is the equivalent stiffness of the soft foot, and t is time. The equation can be solved as ignoring the tedious derivation process to obtain:

$$y(t)=A\sin(\omega t-\alpha) \tag{4}$$

where α is the phase difference between displacement response and exciting force, A is the amplitude of the RFH-module, and can be obtained as:

$$A=\frac{md\omega^2}{\sqrt{(k-M\omega^2)^2+(c\omega)^2}} \tag{5}$$

The RFH-module will leave the ground when the amplitude A is greater than its static deformation and overcomes the gravity of the elastic foot $m_f g$:

$$\frac{md\omega^2}{\sqrt{(k-M\omega^2)^2+(c\omega)^2}} \geq \frac{(M+m+m_f)g}{k} \tag{6}$$

The solution of the inequality can be obtained by ignoring the effect of damping and discarding invalid solution:

$$\begin{cases} \omega \geq \omega_c \\ \omega_c = \sqrt{\dfrac{k(M+m+m_f)g}{(M+m+m_f)Mg+kmd}} \end{cases} \tag{7}$$

where ω_c is the critical value of the switching between two locomotion mechanisms. It means that the module will switch to friction locomotion if the rotary speed is larger than the critical value, while it will switch to inertial locomotion if opposite.

Note S4. Flow field of rigid vibrating block on water surface

The flow field on water surface of a rigid block is a typical case of flexible plate with no deformation and it has been studied in some research. We also carry out some experiments in the same conditions to provide a reference for the comparison with the flexible plate.

As shown in Figure S3a, when the bottom of the block is immersed in water, the water climbs upwards, and a meniscus adheres to the sidewall due to the surface tension. The boundary layer will form in the adhesion area as the block starts to vibrate in vertical direction, and energy is transmitted to the fluid due to the viscous shear force. Oscillation occurs at the meniscus and is transmitted from boundary layer into the fluid, thus a uniform plane traveling wave is formed (Figure S3b). Obliquely transmitted surface waves are also generated due to the sharp corners at two ends, and they superimpose in the middle part resulting larger amplitude than two ends. Energy is transferred to the fluid in the horizontal direction as wave traveling, and eventually a relatively steady outward flow is formed in the middle area.

The wave front will break due to the modulation instability as the vibration amplitude increases to the threshold, and a stable cross wave will generate, as shown in Figure S3c. The adhesion layer attached to the block will rupture and form a surface tension standing wave at sidewall, and the fluid clusters near the wall will move irregularly parallel to the wall under the action of the standing wave. The water far from the wall will compensate due to the conservation

of mass, which leads to the direction of water flow changes suddenly from outward to inward (Figure S3d). Thus, the change of flow under high voltage and large amplitude means that the rigid block cannot generate a sufficient backward water flow and is inappropriate to be used as locomotion component directly.

Note S5. Hardware of the control system

The control system is designed to achieve remote control through a host controller and realize various motion forms. The entire control system consists of a core control system including a host controller, a terminal controller and a communication interface, and a peripheral control system including a motor driver and a power supply module, as shown in Figure S4a. All components of the control system are integrated into the robot, except the host controller.

The task of the host controller is to give commands by operator and display the status of robot, while the terminal controller is responsible for receiving and parsing commands from host controller and also receiving signals and feeding back to the host controller from power supply and motor driver. The terminal controller needs 4 PWM channels to control the motor, 5 ADC channels to monitor the robot state, and 1 USART serial port to communication with Bluetooth low energy (BLE) module. So, the MCU of STM32F103C8T6 is selected for its small size, with standby current of 10 μA and input voltage of 3.3 V–6 V. It can meet all the requirements for the control system and has good support for the subsequent function expansion. The communication module is mainly responsible for two-way information transfer. The desired control distance of this robot is about 20 m within the scope of proximity communication. The BLE method is the most suitable communication solution considering the small transmission data and low power consumption requirement. The selected BLE module has a rated voltage between 3.0 V–3.6 V and a normal operating current of 9 mA, the transmission rate can reach 1 Mbps, and the reference distance is about 60 m.

The locomotion system includes a pair of coreless eccentric motors and a 2-way DC motor driver board as the driving circuit to control the speed of motors through PWM signal. The rotary speeds of the eccentric motor at different voltages are shown in Figure S4b; the speed directly determines the vibration strength and the vibration frequency. A motor with size of Φ6 mm × 12 mm is selected, and the rotary speed is about 14000 RPM at 3.3 V. The motor driver is DRV8833 with two full H-bridges for two-way driving capability; the input voltage of the driver

is between 2.7 V–10.8 V, and one single H-bridge can output a full-scale current of 1.5 A, which meets the driving requirements of two eccentric motors.

Note S6. Power supply analysis

The power supply module mainly includes a rechargeable lithium battery and a switch. The main indexes of battery that may influence the motion performance include capacity, discharge rate, and internal resistance.

The battery should have an appropriate capacity to guarantee the endurance requirements, while its capacity should also be proportional to the internal volume of robot to satisfy the size requirements. So, there should be a compromise between size and capacity. The current consumption of each component in the circuit is measured to evaluate the endurance, as shown in Table S2. At the voltage of 3.0 V, the current consumption of one eccentric motor is about 134 mA with a power of 0.4 W, while the consumption of the motor driver is negligible. The power consumption of the STM32 MCU is about 30 mA with little fluctuation at different voltages. The power consumption of BLE is only 7 mA and also stable at different communication states. It can be seen from the table that the power consumptions of each component satisfy the linear superposition relationship as the modules are connected in parallel in the circuit. Thus, the standby power consumption of the whole control system is 39 mA at voltage of 3.0 V, and the power consumption reach to 282 mA when all modules work together. A 200 mAh rechargeable lithium battery is selected as the power source by comprehensive consideration of the size and endurance requirements. The end voltage of battery will gradually decrease with the capacity decreases, as shown in Figure S4c; and there will also be a drop of end voltage for internal resistance according to $U=E\text{-}Ir$. The internal resistance of the selected battery is about 150 mΩ, then the voltage drop is only 0.045 V when the current is 300 mA. If the power supply is expected to work normally at 3.0 V, then the open circuit voltage is expected to be above 3.2 V considering the energy loss in circuit, which is about 80% of the total capacity. Then the actual working time can reach about 30 minutes theoretically in this condition (with actual working time of 25 minutes in experiment).

Moreover, the discharge capacity of the battery is supposed to meet the required current to ensure normal power supply. The battery for this robot needs to output current of near 300 mA continuously at 3.0 V, while the capacity of the battery is about 200 mAh. So, a power battery with high discharge capacity of 20 C is selected to ensure the actual capacity and safe discharge.

Note S7. Software of the control system

The software of the control system includes the program design for both terminal controller and host controller. The terminal control program is mainly divided into three parts. The first part receives and parses the commands from the host controller through BLE module. If the received commands conform to the preset communication protocol, the commands are passed to the second part, which controls the RFH-module to generate actions according to the received commands. The third part collects the voltages at the ends of the motors and the power supply in real time and feedbacks to the host controller.

The host controller of the control system has two main functions: one is to achieve the connection and communication with the BLE module; the other is to realize command delivery and information reception from terminal controller and display it on the interface. The host controller program is designed in C#, where the UWP API is called in the WinForm application to realize the communication via GATT of BLE. The commands are formatted using self-defined communication protocol to ensure the specification and stability of the transmitted commands (Table S3). The operation logic is basically a sequential structure, and all the operation messages are shown in the Information List panel (Figure S5a). The instructions are encapsulated inside the control buttons and the status information are displayed in the State of Robot panel in real time (Figure S5b).

Note S8. CoT evaluation of the robot

According to the current consumption under different voltages in Table S2, the current of the whole system is about 282 mA at 3.0 V, so that the power consumption of the whole system is about 0.846 W when moving at full speed. Then, the cost of transport (CoT) of the robot can be calculated by:

$$\mathrm{CoT} = \frac{P}{mgv} \tag{8}$$

where P is the instantaneous power consumption, m is the weight of the robot, g is the gravitational acceleration, and v is the robot speed. The CoT of this robot is about 3.0 on the ground and about 14.4 in the water.

Most researches about amphibious robots do not provide the information of CoT, and a brief comparison is shown in Figure S7. The CoT of the robot on ground is moderate compared to

existing amphibious robots, while the CoT of the robot in water is not as satisfying as terrestrial motion, but still in a normal range. The improvement of the motion speed and efficiency of the robot will be an important direction of subsequent research.

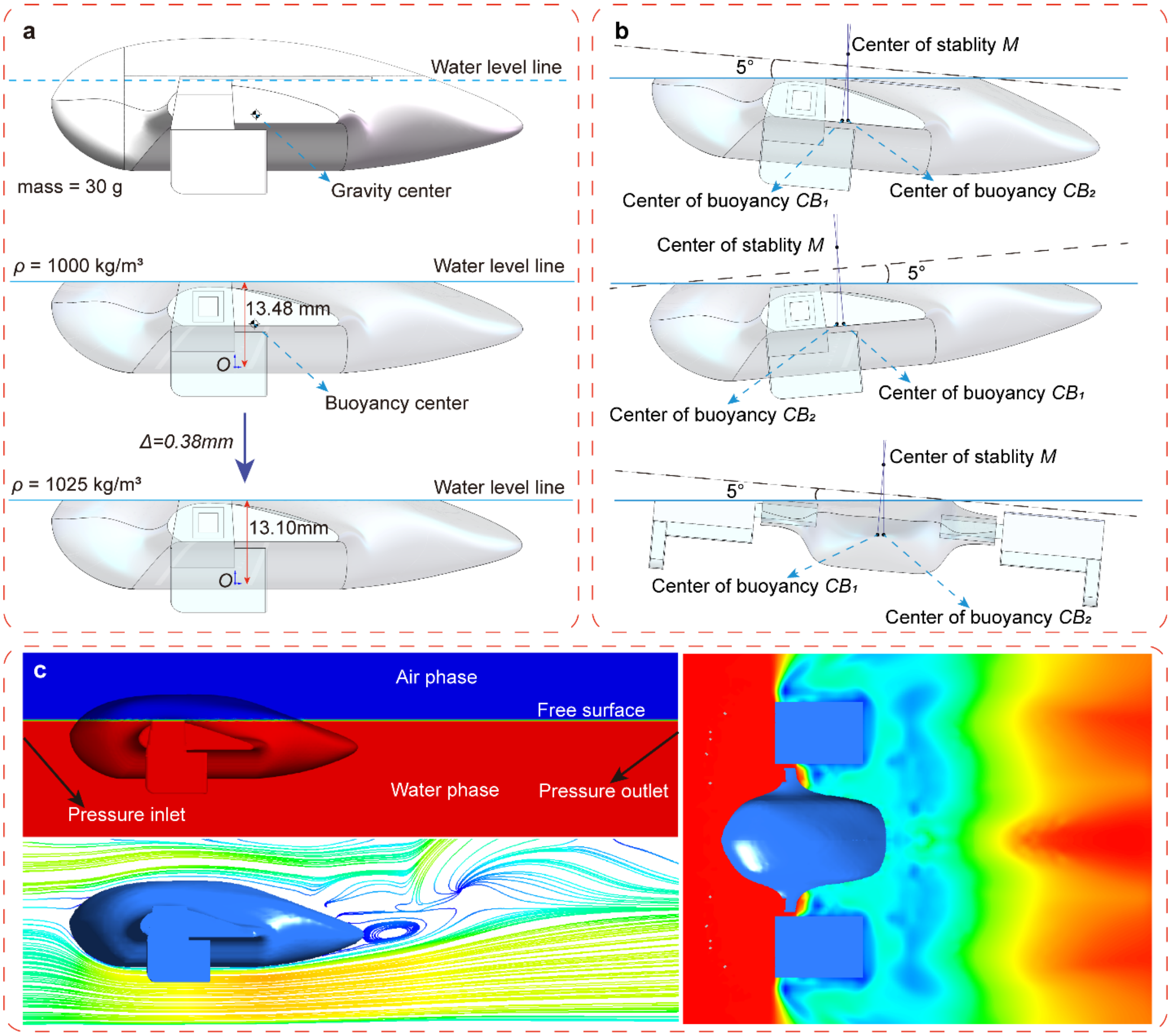

Figure S1. Structure modification for effective aquatic motion and resistance analysis. a) Position adjustment of the water level. b) Motion stability analysis on water surface. (Forward titled stability, backward titled stability, and lateral titled stability) c) Resistance analysis on water surface.

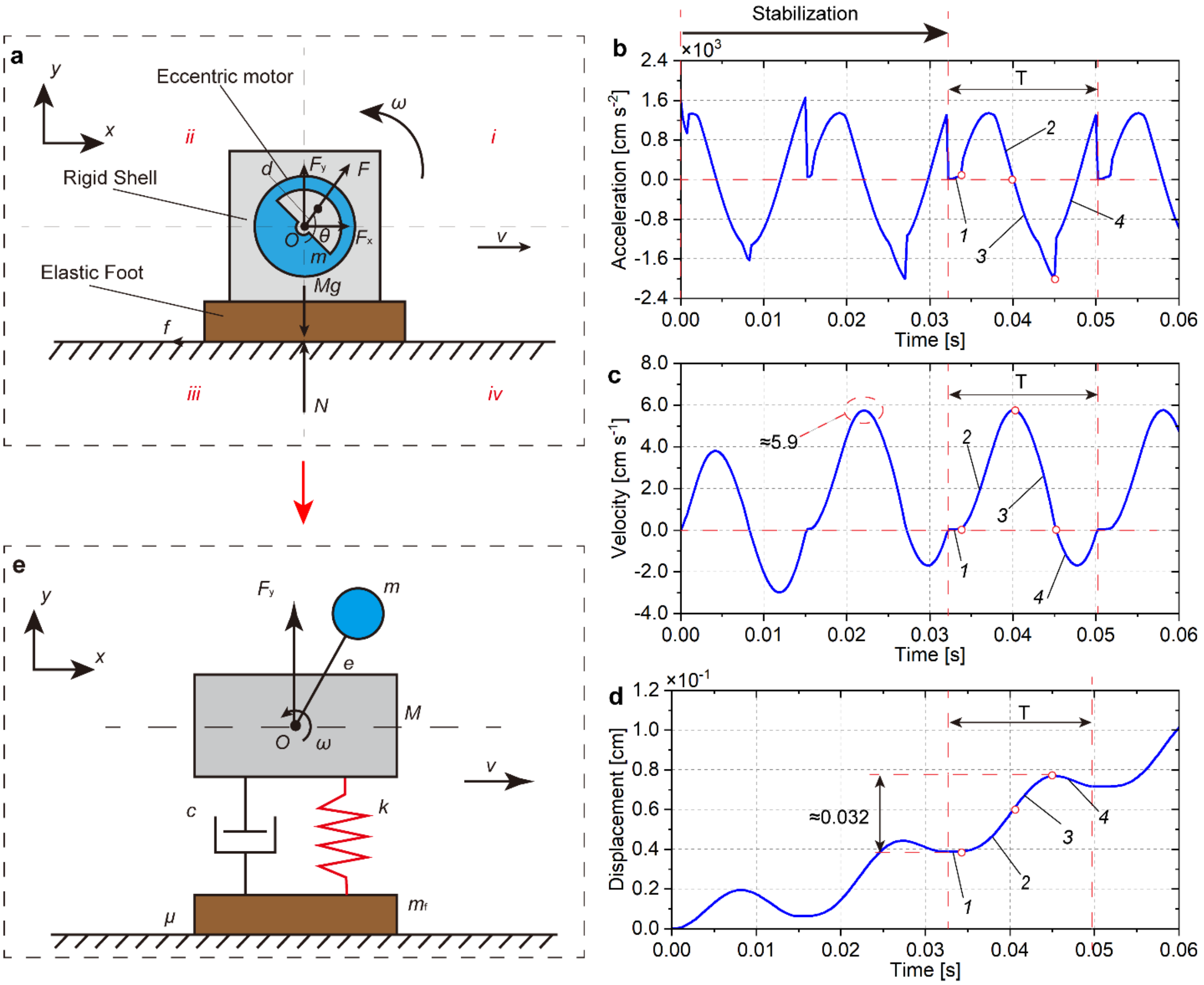

Figure S2. Inertial locomotion mechanism and motion curves of RFH-module. a) The inertial locomotion mechanism diagram. b) The acceleration curve from simulation results. c) The velocity curve from simulation results. d) The displacement curve from simulation results. e) The switch condition analysis between two locomotion mechanisms.

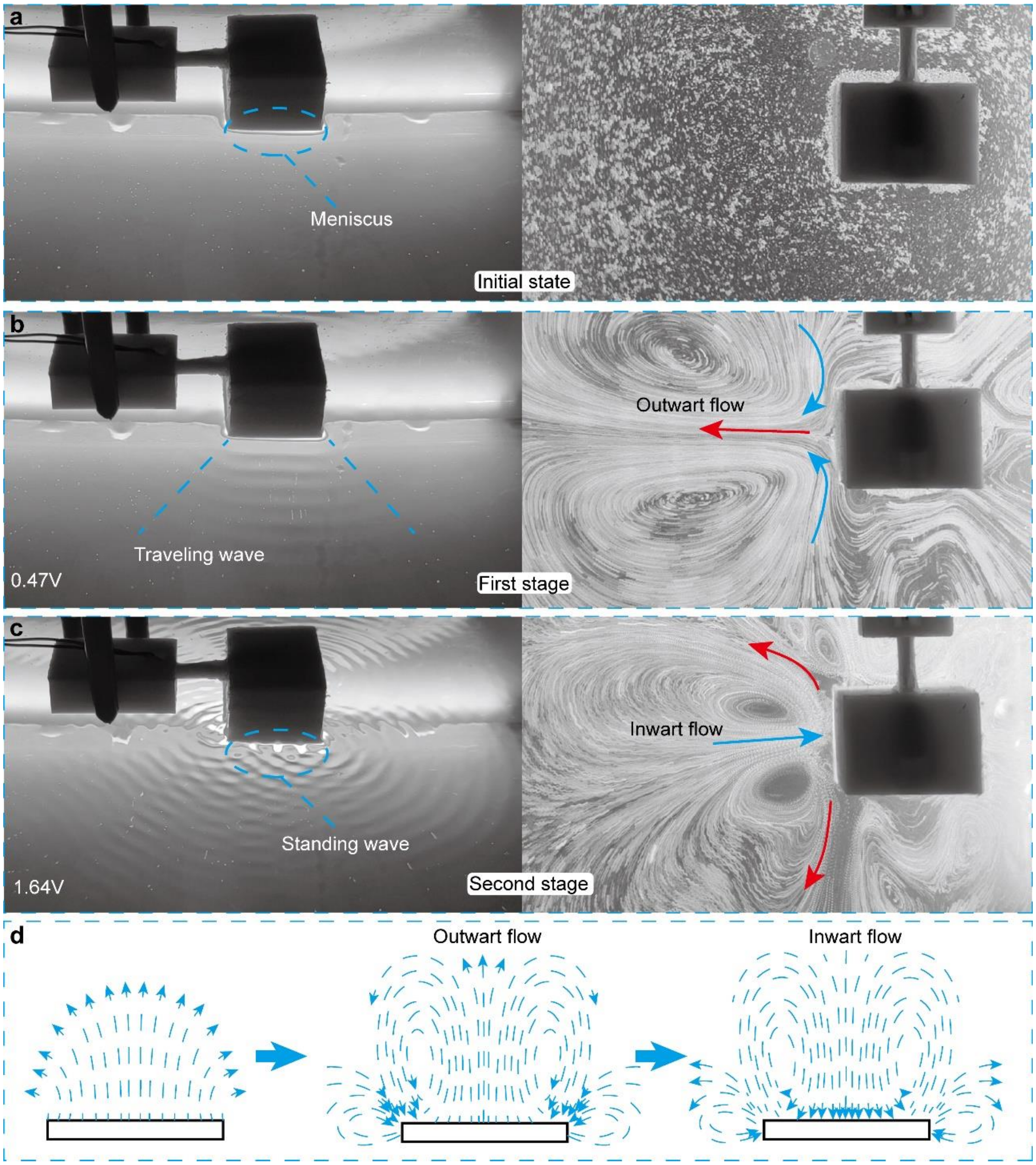

Figure S3. Surface morphology variants of rigid block under different voltages. a) Water surface at rest. b) Water surface flow at first stage. c) Water surface flow at second stage. d) The flow field development process diagrams.

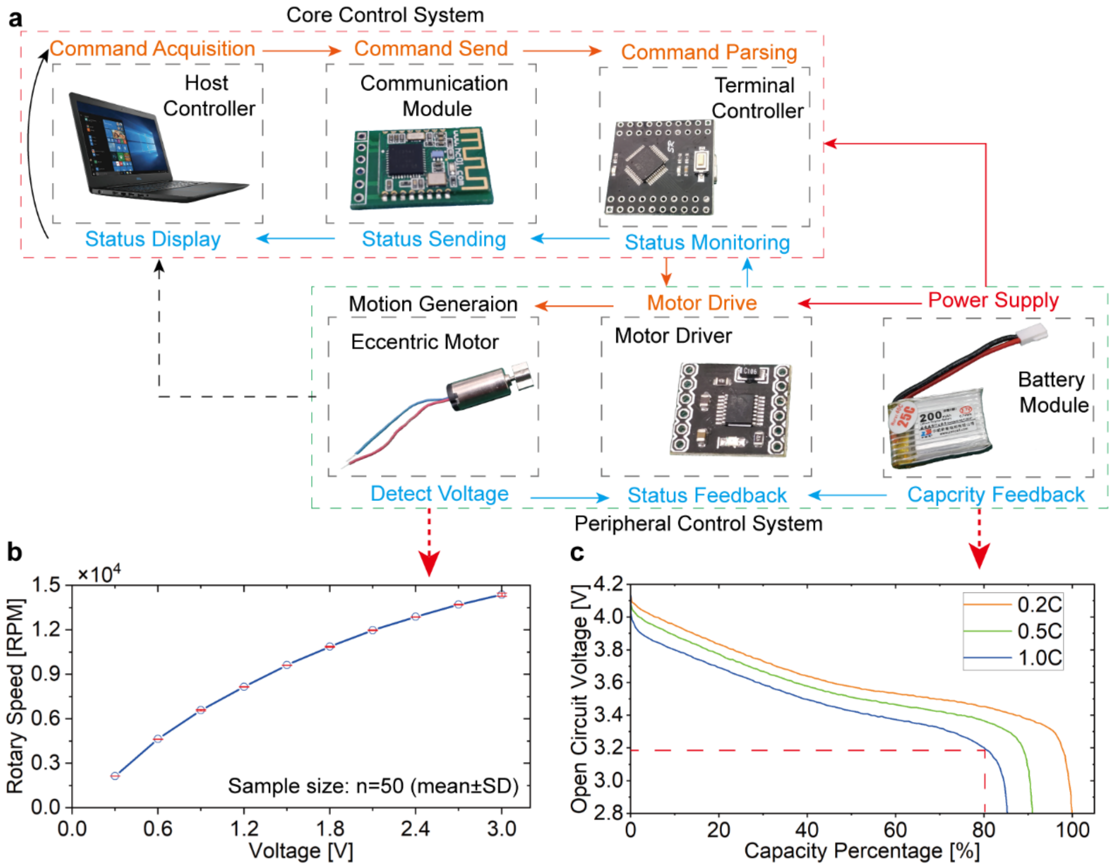

Figure S4. Hardware design of the control system and the tested curves of eccentric motor and battery. a) Module relationship of the control system. b) Rotary speed of eccentric motor at different voltages. c) Discharge curve of the battery.

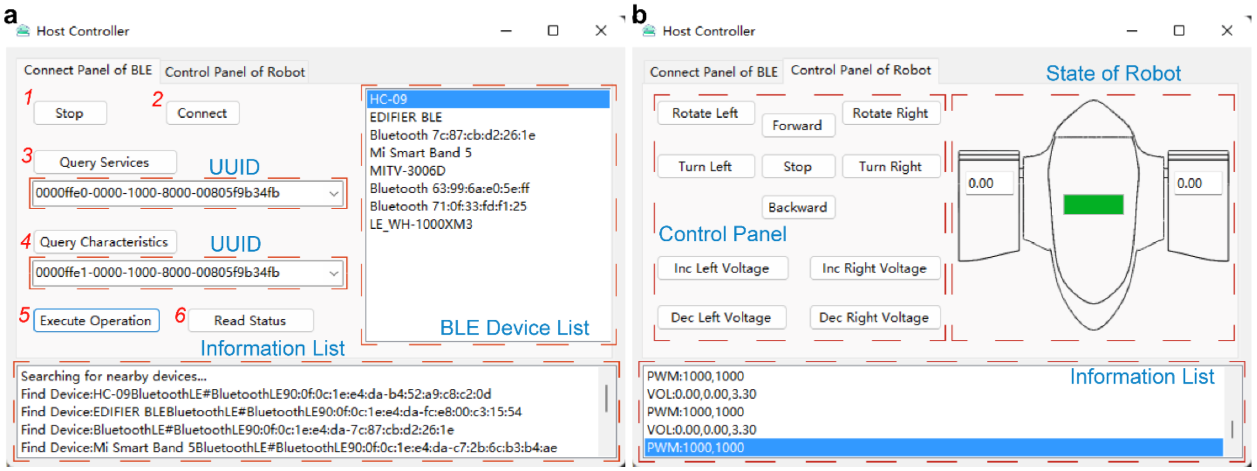

Figure S5. UI design of the software on host controller. a) UI design of the connect panel. b) UI design of the control panel.

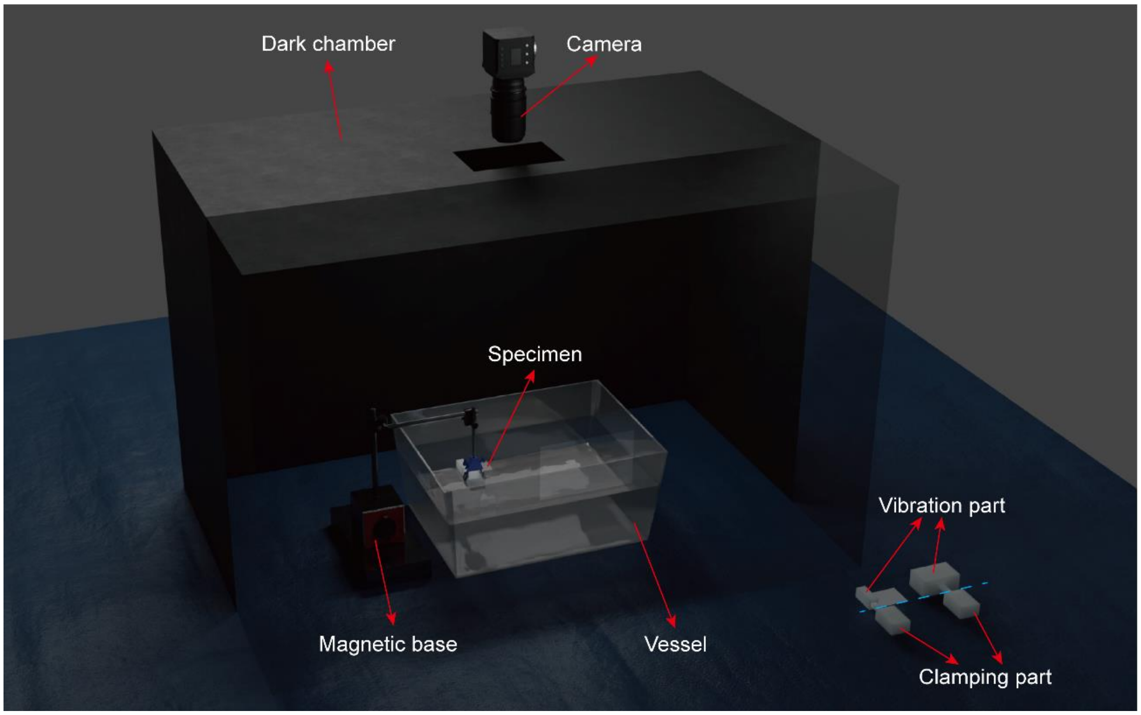

Figure S6. Experiment scene for aquatic locomotion mechanism.

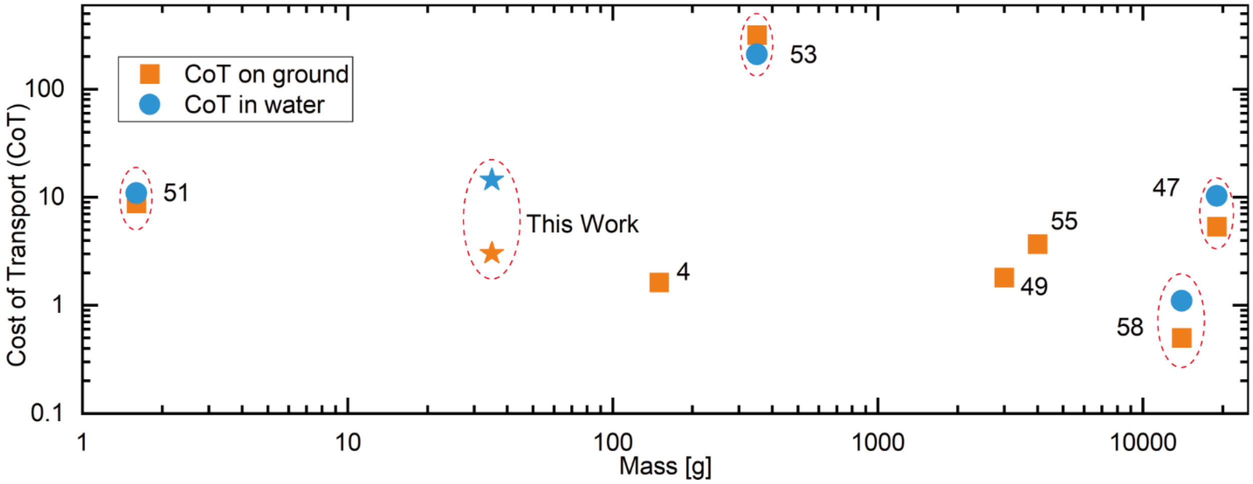

Figure S7. CoT comparison of the proposed amphibious robot with some other amphibious robots.

Table S1. Parameters of the electronic components.

Electronic Components	Main Size [mm]	Mass [g]	Density [g cm^{-3}]
LP602025 200mA Power Battery	27.35×19.70×6.98	6.5	1.79
Coreless Eccentric Motor	Φ6×12.10<br>(Rotor: 3.85×5.64×4.23)	2.4	5.94
DRV8833 DC Motor Driver	18.78×15.79×1.68	1.0	1.94
HC-09 BLE Module	15.64×24.03×3.43	1.9	2.22
Self-locking Six-legged Switch	5.95×5.73×9.98	0.3	1.48
SR-STM32F103C8T6-4 Core Board	25.90×25.40×1.56	2.7	3.32

Table S2. Current consumption under different voltages.

Electronic components/systems	Current consumption [mA]		
	3.0 V	3.3 V	3.7 V
6×12 Eccentric Motor	134	177	202
Motor Driver	0	0	0
BLE (Standby)	7	7	8
STM32 MCU	28	29	29
STM32 MCU + BLE (Standby)	36	38	39
STM32 MCU + BLE (Connected)	36	37	39
STM32 MCU + BLE (Sending)	36	38	39
Whole Control System (Standby)	39	40	41
Whole Control System (Forward State)	282	305	347
Whole Control System (Rotational State)	167	181	202

Table S3. Communication protocol design.

Command	Send Message
Forward	*COM_FORE#
Backward	*COM_BACK#
Turn Left	*COM_TNLT#
Turn Right	*COM_TNRT#
Stop	*COM_STOP#
Rotation Clockwise	*COM_ROCW#
Rotation Counterclockwise	*COM_ROCC#
Increase Left Speed	*COM_INLT#
Decrease Left Speed	*COM_DELT#
Increase Right Speed	*COM_INRT#
Decrease Right Speed	*COM_DERT#
